# Structural and Functional Characterization of *Pseudomonas aeruginosa* CupB Chaperones

**DOI:** 10.1371/journal.pone.0016583

**Published:** 2011-01-31

**Authors:** Xun Cai, Rui Wang, Alain Filloux, Gabriel Waksman, Guoyu Meng

**Affiliations:** 1 State Key Laboratory of Medical Genomics, Shanghai Institute of Hematology, Rui-Jin Hospital, Shanghai JiaoTong University School of Medicine, Shanghai, People's Republic of China; 2 Institute of Structural and Molecular Biology at UCL/Birkbeck, London, United Kingdom; 3 Division of Cell and Molecular Biology, Centre for Molecular Microbiology and Infection, Imperial College London, London, United Kingdom; Indian Institute of Science, India

## Abstract

*Pseudomonas aeruginosa*, an important human pathogen, is estimated to be responsible for ∼10% of nosocomial infections worldwide. The pathogenesis of *P. aeruginosa* starts from its colonization in the damaged tissue or medical devices (*e.g.* catheters, prothesis and implanted heart valve *etc.*) facilitated by several extracellular adhesive factors including fimbrial pili. Several clusters containing fimbrial genes have been previously identified on the *P. aeruginosa* chromosome and named *cup* [1]. The assembly of the CupB pili is thought to be coordinated by two chaperones, CupB2 and CupB4. However, due to the lack of structural and biochemical data, their chaperone activities remain speculative. In this study, we report the 2.5 Å crystal structure of *P. aeruginosa* CupB2. Based on the structure, we further tested the binding specificity of CupB2 and CupB4 towards CupB1 (the presumed major pilus subunit) and CupB6 (the putative adhesin) using limited trypsin digestion and strep-tactin pull-down assay. The structural and biochemical data suggest that CupB2 and CupB4 might play different, but not redundant, roles in CupB secretion. CupB2 is likely to be the chaperone of CupB1, and CupB4 could be the chaperone of CupB4:CupB5:CupB6, in which the interaction of CupB4 and CupB6 might be mediated via CupB5.

## Introduction

*Pseudomonas aeruginosa,* a gram-negative, rod-shaped bacterium, is an important opportunistic human pathogen [2]. Recent statistics shows that *P. aeruginosa* is among the top five infective agents in the hospital, especially in the intensive care departments, responsible for nearly 10% of the hospital-acquired infections such as respiratory tract, blood, urinary tract, ear, skin and soft tissue infections [3], [4], [5]. The prevalence of the *P. aeruginosa* infections might stem from two major reasons: low antibiotics susceptibility and high ability to grow in nearly any natural and artificial surfaces. The organism is known as the most frequent colonizer of medical devices (*e.g.* catheters) causing cross infection in hospital and clinics. Consequently, it is not surprising to find that *P. aeruginosa* is the most common cause for ventilator-associated pneumonias [3]. Therefore, colonization of human tissues, abiotic surfaces such as medical devices, and subsequent development into bacterial biofilm play important roles in the pathogenesis of *P. aeruginosa* [6], [7].

The biofilm formation in *P. aeruginosa* are thought be driven by a number of extracellular appendages including flagella, type IV pili and fimbrial pili. Flagella are thought to be the motor driving the organism towards host or abiotic surfaces [8]. Type IV pili are thought be required for a continuous spreading over the surface by promoting cell aggregation and the formation of microcolonies [8]. Furthermore, by using genetic screening, Vallet and collaborators were able to show that *P. aeruginosa* strains, which lack type IV pili, could still promote biofilm formation via fimbrial pili [1]. This had led to the discovery of three different gene clusters encoding three complete sets of chaperone-usher (CU) secretion systems, termed CupA, CupB and CupC [9].

**Figure 1:**
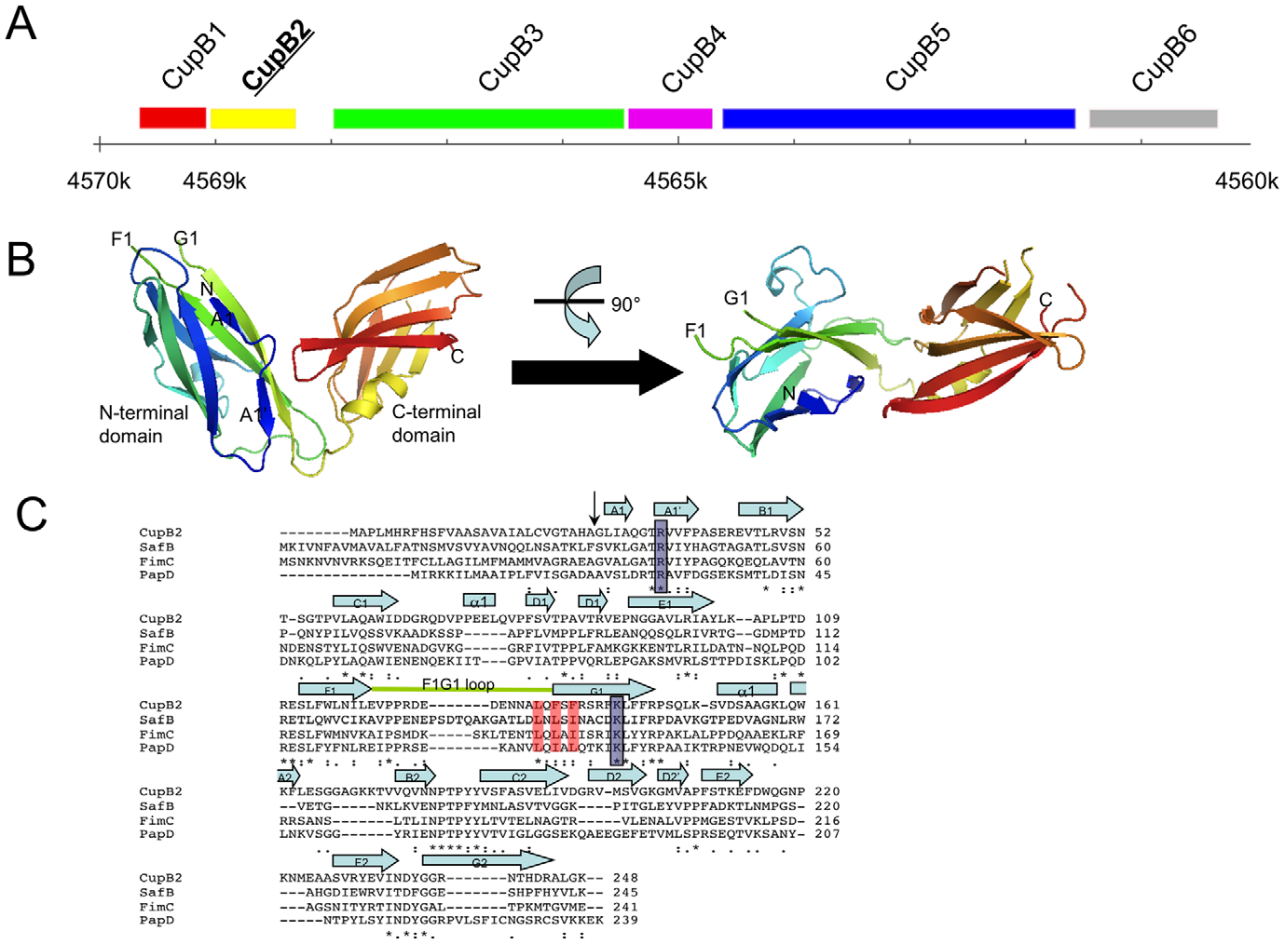
Overall structure of *P. aeruginosa* CupB2 chaperone. A) *Pseudomonas aeruginosa* CupB secretion system. From left to right, their biological functions are thought to be: CupB1 (pili subunit), CupB2 (periplasmic chaperone), CupB3 (outer membrane P-usher), CupB4 (periplasmic chaperone), CupB5 (a Tps-A like protein with unknown functionality), CupB6 (putative adhesin). CupB2 that is crystallographically characterized in this report is highlighted in bold. B) Crystal structure of *P. aeruginosa* CupB2 chaperone. The structure is colored using the rainbow color scheme implemented in Pymol [34] with the N-terminus in blue and the C-terminus in red. C) Sequence alignment between CupB2 and other CU chaperones. “*”, “:” and “.” indicate the strictly, moderately and weakly conserved residues, respectively. The signal peptide is indicated with a vertical black arrow. Secondary structure elements (box for α-helix and arrow for β-strand) are shown above the sequence alignment. F1–G1 loop is highlighted with green line. Strictly conserved Arg36 and Lys141 are highlighted with light blue box with 50% transparency. The alternating hydrophobic residues involved in the strand augmentation between chaperone and pili subunit are colored in red.

The chaperone-usher secretion system is one of the most well characterized bacterial secretion systems in Gram-negative bacteria [10], [11]. Briefly, as implied from its name, the CU system transports its protein cargo, known as pilus subunits, across periplasm and outer membrane in a relay manner mediated by two functionally conserved proteins known as a periplasmic chaperone and an outer membrane usher. The pilus subunit is synthesized as precursor protein containing an N-terminal leader peptide that targets the nascent protein to and across the inner membrane via the Sec machinery. As the pilus subunit is released from the inner membrane, the unfolded polypeptide chain binds to the periplasmic chaperone, first via an interaction between two invariant Arg and Lys residues from the chaperone and the C-terminal carboxylate of the pilus subunit [12]. This crucial interaction induces a protein folding process [12], during which the polypeptide of pilus subunit is folded into an incomplete Ig-like molecule with one crucial β-strand, the 7^th^ strand or G strand of the if-fold, missing. As firstly demonstrated in the PapD:PapK and FimC:FimH structures, the subunit Ig-like fold is completed by receiving in trans a β-strand from its cognate chaperone [13], [14]. The stable chaperone-subunit complex is then recruited to the outer membrane by a highly conserved outer membrane protein, known as the outer membrane usher, for further polymerization with previously-assembled subunits and translocation across the outer membrane [10], [11]. Compared to the conventional CU secretion system [10], [11], the *P. aeruginosa* CupB gene cluster, as shown in Figure 1A, is very different in the following three areas. i) The presence of CupB5, a TpsA-like protein, usually found in two-partner secretion (Tps) systems [15]. Tps systems are specialized secretion systems in Gram-negative bacteria, whereby the protein to be secreted, TpsA, is specifically recognized and transported by its outer membrane-inserted partner, TpsB [16], [17], [18], [19]. The specificity between TpsA and TpsB pairs has been ascribed to two interacting domains, one on TpsA, called the TPS domain, and one on TpsB, the POTRA domain [16]. Besides, PORTA domain is also highly conserved in the core component BamA protein of β-barrel assembly machinery (also known as BAM complex), ensuring the outer membrane proteins' correct folding and insertion into the lipid bilayers [20], [21]. ii) The presence of the *CupB3* gene, which encodes an usher protein containing an N-terminal POTRA domain, is essential for the secretion of CupB5, suggesting that CupB3 is the transporter for CupB5 [15], [16]. iii) Multiple copies of periplasmic chaperones. Based on sequence analysis, two chaperones, known as CupB2 and CupB4, appear required for both secretion of CupB5 and CupB pilus formation. Until now, due to the lack of structural and biochemical data on CupB chaperones, their roles in CupB secretion has remained speculative.

In this study, we report the crystal structure of *P. aeruginosa* CupB2. Similar to other CU chaperones, the CupB2 structure reveals a classical boomerang-like fold consisting of two immunoglobulin (Ig)-like domains. Sequence alignment and structural comparison between CupB2 and other CU chaperones suggest that CupB2 belongs to PapD/FimC chaperone subgroup. Using limited trypsin digestion and strep-Tactin pull-down, we found that CupB2 is likely to be the chaperone of CupB1, but not CupB6. As for CupB4, the result is not that straightforward. CupB4, though sharing significant sequence identity with CupB2, does not show any binding towards the C-terminal carboxylate of CupB1 and CupB6 in limited trypsin digestion and strep-Tactin pull-down assays. This finding was discussed with the existing experimental data of CupB secretion, leading to the hypothesis that CupB4 might be the chaperone of CupB4:CupB5:CupB6, in which the interaction of CupB4 and CupB6 might be mediated via CupB5.

## Results and Discussion

### Overall structure of CupB2

The structure of *P. aeruginosa* CupB2 was determined to 2.5 Å by molecular replacement using SafB and PapD (pdb code: 2CO6 and 1N0L, respectively) as search templates (Figure 1B). The unit cell contains two CupB2 polypeptide chains per asymmetric unit, which were refined independently. The residues 1-28 are predicted to be the signal peptide, and hence are cleaved off upon its translocation across the inner membrane. Consequently, it is not present in the crystal structure of *P. aeruginosa* CupB2. The residues 29–30 in both chains appear to be disordered. Other disordered regions include residues 125–133, 166–173, 217–222 and 248 in chain A and residues 123–136, 165–172, 217–224 and 247–248 in chain B. The structures of the CupB2 chains in the asymmetric unit are very similar with a root mean square deviation (RMSD) in Cα position of 0.3 Å. Size exclusion chromatography of CupB2 is in agreement with a monomeric protein in solution (Figure S1). Hence, unlike the PapD and Caf1M chaperones, there is no self-capping relationship among CupB2 molecules. Therefore, in order to simplify the discussion, these two chains will be treated as identical entities in the following text.

Like other fimbiral chaperones such as *E.coli* PapD, FimC, *Yersinia pestis* Caf1M *etc.*, the crystal structure of CupB2 also reveals a classical boomerang-like fold consisting of two immunoglobulin (Ig)-like domains (Figure 1B). The N-terminal domain contains one short α-helix termed α1 (residue 73–76) and nine β-strands, termed A1–A1′ (residues 31–33 and residues 36–40, for A1 and A1′ strands, respectively), B1 (residues 45–52), C1 (residues 58–65), D1–D1′ (residues 80–83 and residues 86–89, for D1 and D1′ strands, respectively), E1 (residues 92–101), F1 (residues 111–120), and G1 (residues 136–145). Based on the length of the loop between the F1 and G1 strands (F1–G1 loop or FG loop), CU chaperones can be classified into two different subgroups, known as FGL (L stands for “long”) and FGS (S stands for “short”) [22]. Furthermore, as summarized in [22], FGL and FGS also have notable differences in other parts of the structure. With the CupB2 structure available, we now are able to examine its structure against the available chaperone structures, focusing on regions around the A1 strand, the F1–G1 loop and the formation of disulfide bridge: i) CupB2 lacks the extended A1 strand as observed in SafB (Figure 1B and Figure S2). ii) Like PapD and FimC, CupB2 has a short F1–G1 loop (Figure 1C). Furthermore, as shown in SafB and Caf1M, FGL chaperones normally contain five hydrophobic alternating residues in their G1 strands [23], [24]. In comparison, CupB2 has only three hydrophobic residues of this kind (highlighted in red in Figure 1C). iii) Unlike SafB and Caf1M chaperones, CupB2 does not have an FGL-type-conserved disulfide bridge between the F1 and G1 strands. Taking these observations together, we conclude that CupB2 is likely to be an FGS-type chaperone like PapD and FimC.

The CupB2 C-terminal domain is also a typical Ig-like domain containing one α-helix termed α2 (residues 152–159) and eight β-strands, termed A2 (residues 160–163), B2 (residues 175–179), C2 (residues 185–194), D2–D2′ (residues 197–201 and residues 204–206 for D2 and D2′ strands, respectively), E2 (residues 209–214), F2 (residues 227–234) and F2 (residues 237–245). Like the N-terminal domain, the inner core of the C-terminal CupB2 Ig domain is also packed with hydrophobic/aromatic residues such as Leu, Ile Phe, Tyr and Trp. Compared to the N-terminal domain of CupB2, most of the aromatic residues in the C-terminal domain are found in the equator position, leading to a relatively even distribution of hydrophobic residues in the inner core. As a result, the C-terminal domain appears to be more compact than its N-terminal counterpart. Although it is not clear how the hydrophobic residues influence the chaperone activities of these two domains, it is worth to point out that the upper part of the N-terminal Ig domain, where the structures are more flexible and found to be disordered (Figure 1), is important for the chaperone activity. Similar structural feature can also be found in other CU chaperones including PapD, FimC, SafB and Caf1M [13], [14], [23], [24], suggesting that the distribution of hydrophobic residues, particularly the aromatic side-chains, in the inner core might indeed play important structural roles in shaping the two Ig domains into different functional entities.

**Figure 2:**
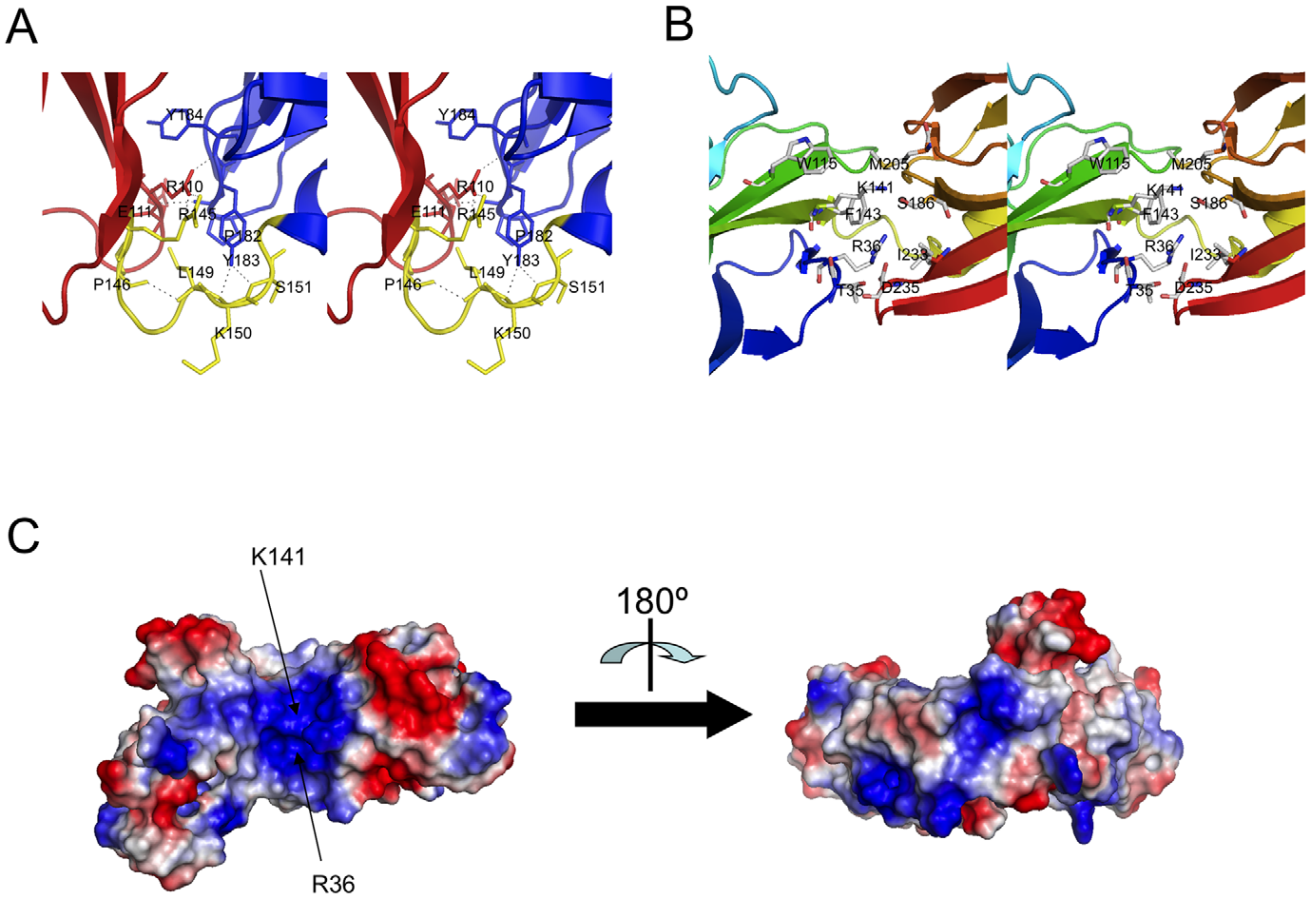
Structural features of *P. aeruginosa* CupB2. A) Polar contacts between the N- and C-terminal Ig domains, colored in red and blue, respectively. The linking loop is colored in yellow. Hydrogen bonds among this region are shown in dash lines. B) The cleft formed by the N- and C-terminal domains. Arg36 and Lys141 lie in the heart of the region. The residues 4 Å away from Arg36 and Lys141 are shown in stick. C) Electrostatic surface of *P. aeruginosa* CupB2 chaperone. The surface is colored according to the electrostatic surface potential (negative charges -59K_B_T in red and positive charges +59K_B_T in blue with linear interpolation in between). Arg36 and Lys141 are located in the heart of the central positively charged pocket, a possible binding site for the C-terminus of the CupB pili subunit. The figure is prepared using Pymol [34].

**Figure 3:**
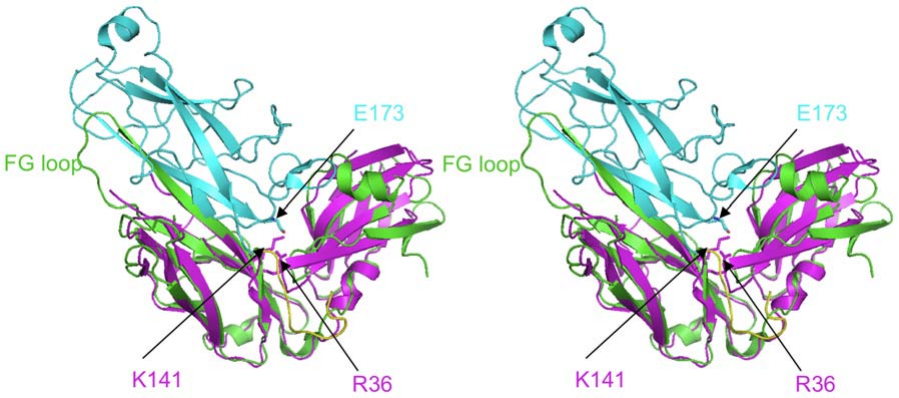
Comparison of *P. aeruginosa* CupB (magenta) and *E. coli* PapD (green) complexed with PapH subunit (cyan). The C-terminal carboxylate of PapH, i.e. E173 shown in stick, is located within a hydrogen bond distance away from Arg36 and Lys141. Furthermore, the F1 strand - loop - G1 strand of CupB2 is superimposed perfectly with that of PapD, mediating the chaperone-subunit interaction.

The N- and C-terminal domains are connected by a kinked linker, residues 145–152 (Figure 2A). The relative orientation of these two domains are stabilized mainly by polar interactions involving the side-chains of Glu111, Arg145, Tyr183 and the main-chains of Arg110, Pro146, Lue149, Lys150, Ser151, Pro182 and Tyr184 (Figure 2A). Interestingly, these residues are all located underneath two invariant positively charged residues, Arg36 and Lys141 lying in the cleft formed by the N- and C-terminal domains (Figure 2C). All the side-chains 4 Å away from Arg36 and Lys141 are shown in Figure 2B. Arg36 and Lys141 are well-conserved residues among PapD-like chaperones [12], where they are involved in the first point of contact with the unfolded subunits exiting the Sec translocon by capturing the emerging C-terminal carboxylate of the subunit's chain. As shown in Figure 3 where CupB2 is superimposed with the structure of the PapD:PapH complex, Arg36 and Lys141, together with the F1–G1 loop, are all in immediate contact with the pilus subunit.

### Interactions between CupB chaperones and its putative binding partners

**Figure 4:**
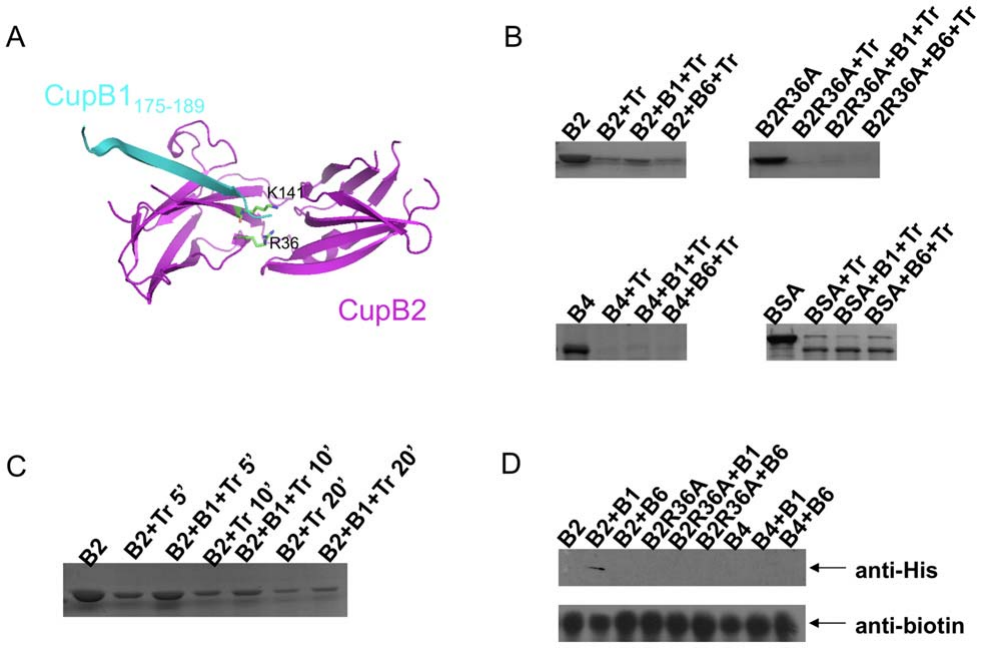
Functional characterization of *P. aeruginosa* CupB chaperones. A) Structural simulation of CupB2 chaperone (magenta) bound to the C-terminus of CupB1 (cyan). Arg36 and Lys141, which are thought to form polar interaction with the C-terminal carboxylate of CupB1, are shown in stick (green). B) Limited trypsin digestion of CupB chaperone upon binding to the C-terminus of CupB1 and CupB6. 3 µg purified protein sample was incubated with 24 times molar excess of CupB1_175–189_ or CupB6_367–381_ for 1 hour at room temperature before the reaction mixture was subjected to trypsin digestion for 10 minutes. SDS-PAGE and Coomassie blue staining were used to monitor the protective effect of CupB1_175–189_ and CupB6_367–381_. C) The CupB2 chaperone was mixed with CupB1_175–189_ as described in (B) and further incubated with trypsin for 5, 10 and 20 minutes, respectively. D) Strep-tactin pull-down assay. 2 µg purified protein was incubated with 3 µg biotinylated peptides (*i.e.* protein:peptide at 1∶24 molar ratio) before the mixture was further mixed with strep-tactin sepharose. The binding of CupB2/CupB4 and CupB1_175–189_/CupB6_367–381_ were monitored by Western blot analysis.

To investigate the CupB chaperones' binding ability towards CupB1 (the presumed major pilus subunit) and CupB6 (a putative adhesin containing an N-terminal adhesin domain and a C-terminal pilin domain), two short peptides corresponding to the C-terminus of CupB1 (CupB1_175–189_) and CupB6 (CupB6_367–381_) were designed based of the structural superimposition between CupB2 and PapD:PapH (Figure 3 and Figure 4A). Two different assays including limited protease digestion and strep-tactin pull-down were used to detect the interactions between CupB2/CupB4 and CupB1_175–189_/CupB6_367–381_.

Kuehn and coworker demonstrated that limited protease digestion could be used to probe the interaction between CU chaperone and the C-terminal peptide of the pilus subunit [25]. We herein also adopted a similar approach to test whether and how CupB2/CupB4 bind to their putative binding partners. The CupB chaperones were first incubated with or without CupB1/CupB6 peptides at 1∶24 molar ratio for 60 minutes followed by trypsin digestion. As shown in the top-left panel of Figure 4B, the presence of CupB1_175–189_, but not CupB6_367–381_, appears to have protective effect on CupB2 against trypsin. To support this finding, limited protease digestions were repeated and monitored at different time points. As shown in Figure 4C, the presence of CupB1 peptide clearly slowed down the proteolytic activity of trypsin upon CupB2 over 5, 10 and 20 minutes. We then further investigated whether the interaction between CupB2 and CupB1 peptide is due to the formation of CupB2:CupB1_175–189_ complex mediated by the conserved positively charged residues, Arg36 and Lys141 (Figure 4A). As shown in the top-right panel in Figure 4B, neither CupB1_175–189_ nor CupB6_367–381_ has any protective influence on CupB2(R36A) mutant, suggesting that the interaction between CupB2 and CupB1 peptide might occur through Arg36. To further confirm this hypothesis, a different technique, i.e. strep-tactin pull-down assay, was used. As shown in Figure 4D, only the biotinylated CupB1 peptide, but not the CupB6 peptide, can pull down the CupB2 chaperone. Consistent with the limited trypsin digestion described above, the CupB1 peptide showed no binding toward the CupB2(R36A) mutant, strongly implying that CupB2 is indeed the periplasmic chaperone for CupB1.

**Figure 5:**
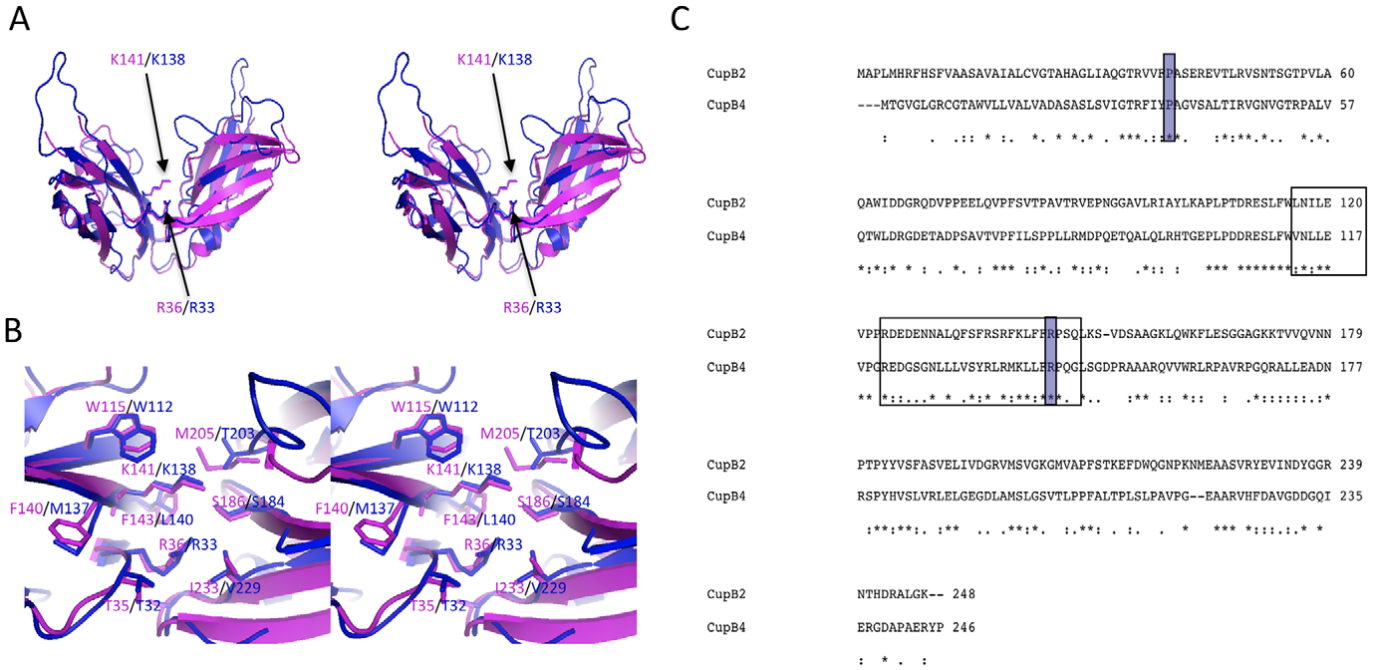
Structural comparison between CupB2 and CupB4 chaperones. A) Homology model of CupB4 (blue) predicted based on *E. coli* PapD and *P. aeruginosa* CupB2 (magenta). B) Enlarged views of the putative active sites of CupB2 (magenta) and CupB4 (blue). Side-chains of the residues that are located 4 Å away from the invariant Arg and Lys are shown in stick representation. C) Sequence alignment between CupB2 and CupB4. Strictly conserved Arg, Lys and F1 strand - loop - G1 strand region, which are thought to be important for chaperone-subunit interaction, are highlighted. “*”, “:” and “.” indicate the strictly, moderately and weakly conserved residues, respectively.

In comparison, the CupB4 chaperone does not bind CupB1_175–189_ or CupB6_367–381_ (Figure 4BD). This is very puzzling as further investigation on the CupB4 sequence suggests that CupB4 is a CupB2-like protein, with little differences in the overall structure and the areas that mediate chaperone-subunit interaction (Figure 5). CupB2 and CupB4 share 32% sequence identity (Figure 5C). By homology modeling, CupB4 is predicted to have two Ig domains with similar F1–G1 loop and relatively conserved active site in the central cleft (Figure 5AB). Furthermore, as shown by Ruer and coworkers, CupB4 is thought to be a critical component in CupB secretion [15]. Deletion of CupB4 gene abolished the surface location of CupB1 and CupB5. This had previously led to the hypothesis that CupB4 might interact with CupB6 adhesin and this interaction, like the chaperon-adhesin complex in other CU systems [26], [27], is essential to activate the outer membrane P-usher for the subsequent assembly of pili subunits, CupB1, and the TpsA-like molecule, CupB5 [15]. However, in this report, as shown in Figure 4, both the limited protease digestion and strep-tactin pull-down assays clearly showed that CupB4 has very weak binding affinity, similar to that of the CupB2(R36A) mutant, against the C-terminus of CupB1 and CupB6 (Figure 4BC), suggesting that CupB4, despite sharing a similar protein fold with CupB2, might utilize a different mechanism, which might require the presence of CupB5, to accompany CupB6. Interestingly, *CupB5* gene is located in between CupB4 and CupB6 in *CupB* gene cluster (Figure 1A), and as shown by Fronzes et al, the sequential expression of a bacterial secretion gene cluster is essential for the formation and the stability of a multi-component complex in periplasm and outer membrane [28].

### Summary

With the structural and biochemical data presented here, it is now clear that CupB2 is an FGS-type chaperone with two functionally different Ig domains. As suggested by the functional studies presented in this report, CupB2 is likely the chaperone of CupB1, but not CupB6 or CupB5. The interaction between CupB2 and C-terminus of CupB1 is likely to be mediated by two invariant residues, Arg36 and Lys141, in the cleft formed by N- and C-terminal Ig domains.

As for the second putative chaperone in the CupB system, the exact function of CupB4 remains speculative. Previously, based on its impact upon the secretion of CupB1 and CupB5, CupB4 is hypothesized to interact with CupB6. Herein, we used limited protease digestion and strep-tactin pull-down assays to show that the CupB4:CupB6 interaction might be a lot more complicated than a simple chaperone-subunit-like association. Our functional studies demonstrated that CupB4, like the CupB2(R36A) mutant, showed very little binding towards the C-terminal carboxylate of CupB6. However, as shown in the previous studies [15], CupB4 is essential for the secretion of CupB5 and the activation of P-usher. Taking these observations together, we hypothesize a ternary complex of CupB4:CupB5:CupB6 in CupB secretion. Admittedly, with the current data, we have no clue how a TpsA-like CupB5 could mediate the interaction of CupB4 and CupB6. Further investigation is needed for a more comprehensive understanding of the CupB chaperones, which, like Pap system [29], [30], might in turn contribute to facilitate the design of novel small molecules that prevent the biofilm formation mediated by fimbrial pili, and more importantly, open a new front to tackle *P. aeruginosa*.

## Materials and Methods

### Bacterial strains, plasmids, and culture conditions

Bacterial strains used in this study include *Escherichia coli* BL21(DE3) (Novagen), *E. coli* DH5α (Life Technologies). *E. coli* strains were grown in Luria–Bertani (LB) agar or in LB broth. For storage, all the bacterial strains are kept at − 80°C in LB containing 50% glycerol. 100 µg ml^−1^ ampicillin was used for plasmid selection.

### Construction of plasmids used in this study

To generate constructs for crystallographic and functional studies, *Pseudomonas aeruginosa* PAO1 genomic DNA was used as a template to amplify the coding sequence for CupB2_1–248_ and CupB4_24–246_, with two *Bsa*I sites at the ends of the forward and reverse primers, respectively. The primers were:

5′-ATGGTAGGTCTCAAATGGCGCCGCTAATGCATCGTTTTC -3′ (forward, CupB2_1–248_),

5′-ATGGTAGGTCTCAGCGCTTTTGCCGAGTGCCCTATCGTGG-3′ (reverse, CupB2_1–248_),

5′-ATGGTAGGTCTCAAATGTCCGCCTCGCTGTCCGTGATC -3′ (forward, CupB4_24–246_),

5′-ATGGTAGGTCTCAGCGCTCGGGTATCTCTCTGCCGGCG-3′ (reverse, CupB4_24–246_),

The resulting PCR fragments encoding CupB2_1–248_ and CupB4_24–246_ were digested with *Bsa*I and ligated into *Bsa*I-digested pASK-Iba33plus (IBA), generating pASK-CupB2_1–248_ and pASK-CupB4_24–246_.

In order to test CupB2 specificity against C-terminus of CupB1 and CupB6, a point mutation in CupB2_1–248_ converting the Arg at position 36 to Ala, primers were generated using the following primers: 5′- TGATCGCACAGGGCACTGCCGTCGTCTTTCC-3′ (forward), 5′- GGAAAGACGACGGCAGTGCCCTGTGCGATCA-3′ (reverse) and the QuickChangeTM site-directed mutagenesis kit (Stratagene) was used. The base mutation responsible for the amino acid is underlined. DNA sequencing was performed to confirm the mutation and plasmid containing the R36A mutation was then transformed into *E. coli* BL21(DE3).

### Expression, purification and crystallization

To purify CupB2, *E. coli* BL21(DE3)/pASK-CupB2_1–248_ grown at 37°C to OD_600_ of 0.5–0.7 and then induced for 4 h using 0.2 mg l^−1^ anhydrotetracycline (IBA) at the same temperature. Following induction, bacteria were centrifuged at 4,000 g for 20 minutes, and cell pellets were resuspended in 20 mM Tris pH 8.0, 20% sucrose and10 mg ml^−1^ lysozyme and incubated at 4°C for 20 min. The periplasmic proteins were recovered by centrifuged at 12,000 g for 20 min. The supernatant, *i.e.* periplasmic extraction, was loaded onto a HisTrap column (GE Healthcare) and eluted with 20 mM Tris, pH 8.0, 500 mM NaCl and 150 mM imidazole. The eluate was pooled and concentrated before it was loaded onto an S100 gel-filtration column (GE healthcare). The peak fraction was estimated to be ∼95% pure, as indicated by Coomassie blue-stained SDS-PAGE.

To purify CupB4, *E. coli* BL21(DE3)/pASK-CupB4_24–246_ were grown and expressed using the same protocol described above. Bacteria were harvested by centrifugation (4,000 g, 20 min). Cell pellets were resuspended in 20 mM Tris, pH 8.0, 150 mM NaCl and sonicated. The clear lysate were recovered by centrifugation (30,000 g, 30 min). The supernatant was then loaded onto a HisTrap column and eluted with 20 mM Tris, pH 8.0, 500 mM NaCl and 150 mM imidazole. The eluate was pooled and concentrated before it was loaded onto an S100 gel-filtration column (GE healthcare). The purity of the peak fraction was monitored by Coomassie blue-stained SDS-PAGE.

For crystallization, purified CupB2 was concentrated to ∼34 mg/ml using an Amicon Ultra 10 concentrator with 10 kDa cut-off (Millipore). CupB2 crystals with dimensions of 0.30 mm ×0.3 mm ×0.25 mm were obtained at room temperature using the hanging-drop vapor diffusion method. The reservoir solution contained 100 mM sodium citrate pH 5.4, 14% (w/v) PEG4000, 10% (v/v) isopropanol. The hanging drop contained a 1∶1 (v/v) ratio of reservoir and protein solutions. Crystals were flash-cooled to 100 K by liquid nitrogen in the presence of 20% PEG 400. Crystals of CupB2 diffracted to 2.5 Å and were in space group *C*2 with cell dimensions a  = 94.2 Å b = 65.8 Å c = 87.6 Å β = 105.9, and two molecules in the asymmetric unit.

### Data collection and phasing

Diffraction data for CupB2 native crystals were recorded on BL17U at Shanghai Synchrotron Radiation Facility (SSRF, Shanghai, China). CupB2 data were integrated and scaled using MOSFLM/SCALA [31]. The statistics of data collection are reported in Table 1.

**Table 1:** Data collection and structure refinement statistics of *P. aeruginosa* CupB2.

**Data collection**		
Derivative		Native
Source/Station^a^		BL17U
Wavelength (Å)		0.9798
Resolution range (Å)		84.1-2.5
Observations (*I/*(*I*) >0)		2738020
Unique reflections (*I*/σ(*I*) >0)		17834
High resolution shell (Å)		2.64-2.50
*R_sym_ (%)* b,c		7.3 (11.7)
<*I*/σ(*I*)>^c^		15.5 (8.0)
Completeness^c^ (%)		98.3 (95.8)
Redundancy^c^		5.7 (5.2)
**Structure refinement**		
Resolution range (Å)		84.1 – 2.5
*R*-factor (%)		19.8
*R*-factor (high resolution shell)^d^		30
*R*_free_ (%)^e^		25.9
*R*_free_ (high resolution shell)		36.9
Total number of non-hydrogen atoms		
Protein atoms		2946
Water molecules		194
R.m.s. deviations:^f^		
Bond length (Å)		0.019
Bond angle (°)		1.757
Main chain *B*-factors (Å^2^)		0.8
Side chain *B*-factors (Å^2^)		1.981
Wilson *B*-factor (Å^2^)		48.3
Average *B*-factor protein atoms (Å^2^)		21.3
Average *B*-factor solvent atoms (Å^2^)		27.5

a Beamline designations refer to the Shanghai Synchrotron Radiation Facility, Shanghai, P. R. of China.

b *R*_sym_ = ∑(*I*-<*I*>)^2^/∑*I* ^2^.

c overall, high resolution shell in parentheses.

d high resolution shell: 2.64- 2.50 Å.

e *R*_free_ calculated using 5% of total reflections omitted from refinement.

f R.m.s. deviations report root mean square deviations from ideal bond lengths/angles and of *B*-factors between bonded atoms [35].

CupB2 was phased by molecular replacement using *Samonella enterica* SafB (pdb code: 2CO6) and *E. coli* PapD (pdb code: 1N0L) as search models. To prepare the search models, CupB2 sequence was firstly aligned with SafB and PapD sequences using ClustW2 (http://www.ebi.ac.uk/Tools/clustalw2/), respectively. The resulted sequence alignment was supplied to program CHAINSAW [31] to prune the non-conserved residues, i.e. residues that differ in CupB2 and SafB/PapD were changed to alanine. The pruned models derived from SafB (2CO6) and PapD (1N0L) structures were then served as search templates in PHASER [31]. Refmac5 and PHENIX.REFINE, together with intermittent manual building in COOT were used to correct and improve the initial model produced by PHASER. CupB2 residues 29–30, 125–133, 166–173, 217–222 and 248 appear to be disordered, hence the electron density map for these residues are not available for model building.

### Structure refinement

The structure of CupB2 was refined by conjugate gradient minimization (REFMAC5) [31] with intermittent manual rebuilding, refining individual *B*-factors applying a TLS correction (2 TLS group, 40 parameters) [32]. The final model of CupB2 contains residues 31–247 and 194 water molecules. Ramachandran statistics (PROCHECK) [33] on the CupB2 structure indicate that 95.9 percent of the atoms are in the most favored region, and 4.1 percent are in the additionally allowed regions. The detailed structure refinement statistics are reported in Table 1. Coordinate of CupB2 has been deposited into the Protein Database Bank (entry code 3Q48).

### Peptide synthesis and purification

In the functional studies of the CupB chaperones, the peptides were all purchased from GL Biochem. The peptide was synthesized in the solid phase and then purified to >95% purity by high-performance liquid chromatography. The molecular mass of the peptide was confirmed by mass spectroscopy. In order to generate peptide for pull-down assay, N-terminal biotinylated peptides were also synthesized. Distilled water was used to dissolve these CupB1 and CupB6 peptides. 100% DMSO were used for their biotinylated derivatives.

### Proteolytic characterization of CupB chaperones

3 µg purified CupB2, CupB2(R36A) and CupB4 was incubated with 24X molar excess of CupB1 peptide (NH_2_-AGTGLSRIRYLLAYE-COOH, CupB1_175–189_), CupB6 peptide (NH_2_-AGVADGAAEFTFTFP-COOH, CupB6_367–381_) for 1 hour at room temperature before 1 µg trypsin was introduced to the mixture. The final protein mixture (5 µl) was then further incubated at 37°C for 10 min before the enzymatic reaction was stopped by adding SDS-loading buffer and boiling. 5 µl of digested samples were applied to 15% SDS-PAGE followed by Coomassie blue staining.

### Pull-down assay using Biotin-labeled peptides

2 µg purified CupB protein was incubated with 3 µg biotinylated peptides (CupB1_175–189_, CupB6_367–381_) for 1 hour at room temperature, respectively. Each protein sample was then further incubated with 40 µl 50% (w/v) strep-tactin sepharose solution (IBA) for 1 h. Strep-tactin resin was harvested by centrifugation and rinsed with buffer containing 20 mM Tris, pH 8.0, 500 mM NaCl, 1 mM EDTA three times. To harvest the protein, each sample was heated and centrifuged. For Western blot analysis, proteins were resolved by SDS-PAGE using 15% polyacrylamide gels and transferred to PVDF membrane. To ensure that comparable amounts of protein were analyzed, similar volumes from different protein:peptide mixtures were loaded into each lane. The blots were blocked with 5% defatted milk, probed with anti-His (Tiangen Biotech) and HRP linked anti-biotin antibodies (Cell Signaling Technology). Immunocomplexes were visualized by chemiluminescent (Pierce).

## Supporting Information

Figure S1 **Crystal packing and gel filtration characterization of CupB2 fail to identify a self-capping relation of CupB2 in solution.** A) Crystal packing of CupB2. B and C) Preliminary characterization of CupB2 (24.3 kDa) using an analytical gel filtration chromatography, S12. Ovalbulmin (44 kDa) and Ribonuclease A (13.7 kDa) were obtained from GE healthcare and used as standard protein markers in gel filtration.
(TIF)

Figure S2 **Structural superimposition between CupB2 (magenta) and SafAB complex (cyan and green respectively).** The extended A1 strand from SafB is labeled.
(TIF)
