# Supplementary figures and images for: Structural and Functional Characterization of Pseudomonas aeruginosa CupB Chaperones
Source: PLoS One. 2011 Jan 31;6(1):e16583. doi: 10.1371/journal.pone.0016583 (PMC3031594; doi:10.1371/journal.pone.0016583)

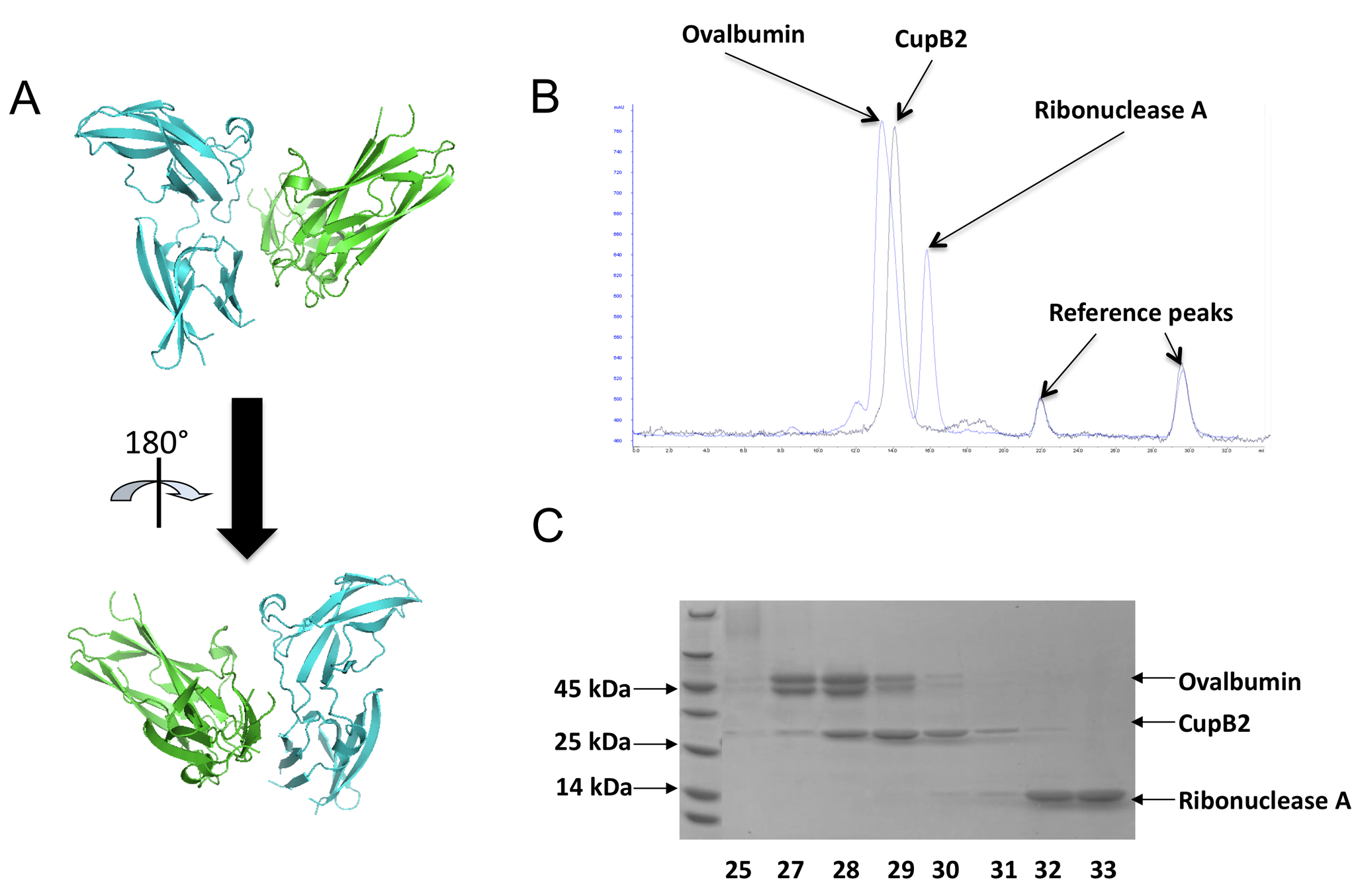

Supplement: Figure S1 — Crystal packing and gel filtration characterization of CupB2 fail to identify a self-capping relation of CupB2 in solution. A) Crystal packing of CupB2. B and C) Preliminary characterization of CupB2 (24.3 kDa) using an analytical gel filtration chromatography, S12. Ovalbulmin (44 kDa) and Ribonuclease A (13.7 kDa) were obtained from GE healthcare and used as standard protein markers in gel filtration. (TIF) [file pone.0016583.s001.tif]

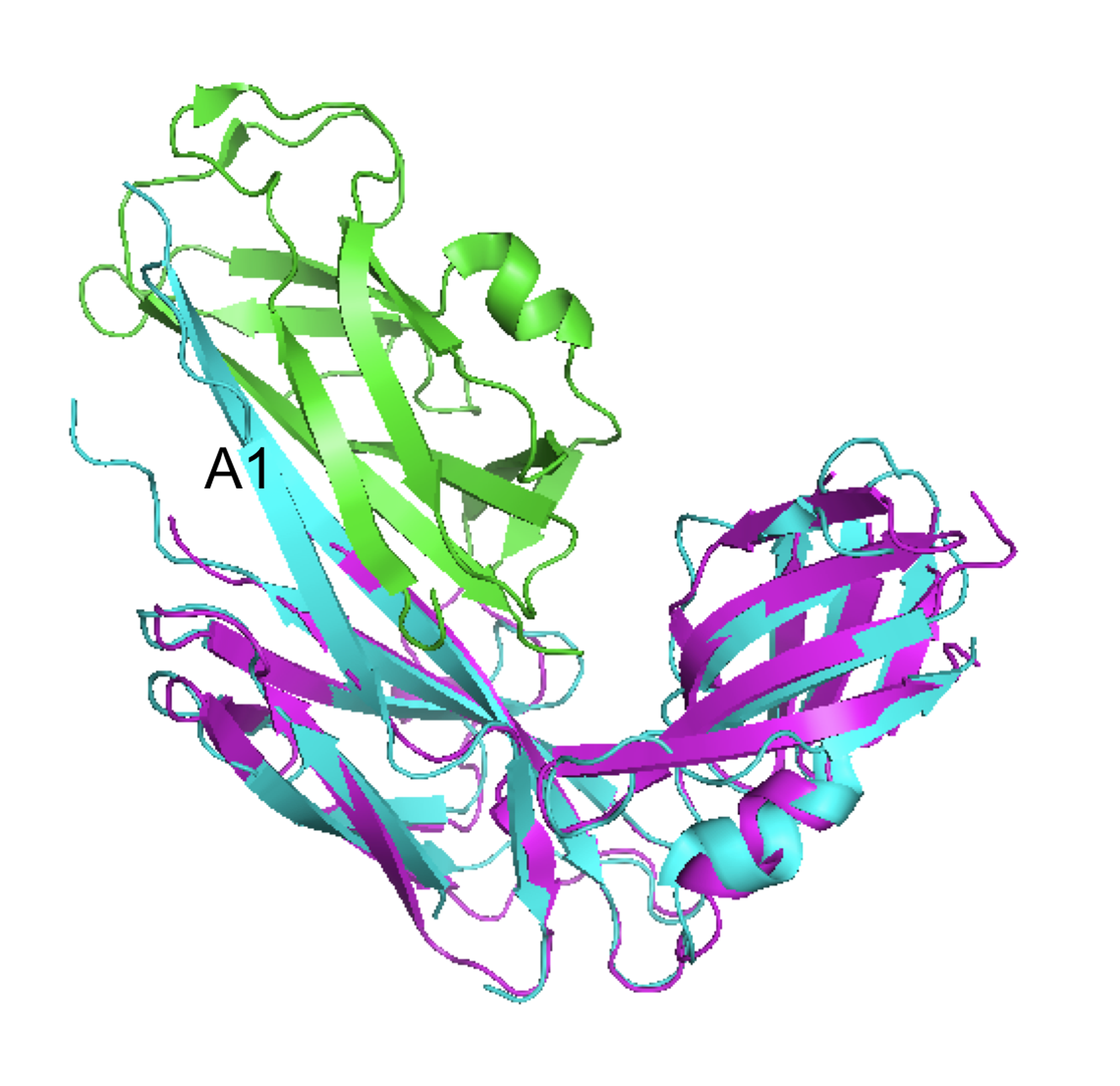

Supplement: Figure S2 — Structural superimposition between CupB2 (magenta) and SafAB complex (cyan and green respectively). The extended A1 strand from SafB is labeled. (TIF) [file pone.0016583.s002.tif]
